# Emergency right hemicolectomy for inflammatory cecal masses mimicking acute appendicitis

**DOI:** 10.1186/1749-7922-9-7

**Published:** 2014-01-20

**Authors:** Hakan Guven, Bora Koc, Fazil Saglam, Irem Akin Bayram, Gokhan Adas

**Affiliations:** 1 Department of Surgery, Okmeydanı Training and Research Hospital, İstanbul, Turkey; 2 Faculty of Medicine, Department of Radiology, İstanbul University, İstanbul, Turkey; 3 Department of Surgery, Başkent University, Oymacı sokak No: 7 Altunizade, İstanbul, Turkey

**Keywords:** Appendicular mass, Right hemicolectomy, Ileocecal resection

## Abstract

**Background:**

Unexpected inflammatory cecal masses of uncertain etiology, encountered in the emergency surgical departments can be indistinguishable, and appropriate operative management of these cases is a dilemma for the surgeons.

**Methods:**

Over a 30-months period between January 2009 and June 2011, a series of 3032 patients who live in sub-urban underwent emergency surgery for clinical diagnosis of acute appendicitis and ileocecal resection or right hemicolectomy for inflammatory cecal mass were performed in 48 patients.

**Results:**

28 men and 20 women from suburban between ages 16–73 presented with right iliac fossa pain. The major presenting symptom was pain in the right iliac fossa (100%). On physical examination; tenderness at or near the McBurney point was detected in 44 (91,6%) patients. The range of the leucocyte level was between 8.000 to 24.000 and mean level is 16.000. After initial laparoscopic exploration, ileocecal resection or right hemicolectomy was performed conservatively because of the uncertainty of the diagnosis. Overall 32 patients underwent ileocecal resection and 16 patients underwent right hemicolectomy. Pathology revealed appendicular phlegmon in 18 patients, perforated cecal diverticulitis in 12 patients, tuberculosis in 6 patients, appendiceal and cecal rupture in 4 patients, malign mesenquimal neoplasm in 4 patients, non-spesific granulomatous in 2 patients and appendecular endometriosis in 2 patients.

**Conclusion:**

Most inflammatory cecal masses are due to benign pathologies and can be managed safely and sufficiently with ileocecal resection or right hemicolectomy. The choice of the surgical procedure depends on the experience of the surgical team.

## Introduction

Appendectomy for appendicitis is the most commonly performed emergency operation in the world. Compared with younger patients, elderly patients with appendicitis often pose a more difficult diagnostic problem because of the atypical presentation, expanded differential diagnosis, and communication difficulty. These factors contribute to the disproportionately high perforation rate seen in the elderly [1].

An appendiceal mass is the end result of a walled-off appendiceal perforation and represents a pathological spectrum ranging from phlegmon to abscess [2]. This condition is a common surgical entity, encountered in 2%-6% of patients presenting with acute appendicitis [2-4]. It has been suggested that delays in presentation are responsible for the majority of perforated appendices or the other complications.

Malignancy and appendiceal inflammation frequently form masses which are virtually indistinguishable and surgeons are often challenged to determine the pathologic origin of masses [5]. There are many reports in the literature that have addressed this promiscuousness, and right hemicolectomy has been recommended because of the concern of possible malignancy [5-8]. The studies were carried out to evaluate the pathologies and surgical management of the inflammatory cecal masses in patients with suspected appendicitis. In this study, we aim to present the diversity of the inflammatory cecal masses mimicking acute appendicitis.

## Methods and results

A series of 3032 patients from suburban who underwent emergency surgery for clinical diagnosis of acute appendicitis at Bagcılar Training and Research Hospital and Okmeydanı Training and Research Hospital between January 2009 and June 2011 were evaluated retrospectively. 48 patients who had right-hemicolectomy or ileocecal resection for inflammatory cecal masses of uncertain etiology were included in our study. Right-hemicolctomy was performed as formal resection of the right colon including lymphatic drainage along the ileocolic and right colic arteries. The relevant case notes were subsequently retrieved from the medical records and the following data were obtained for each patient: age, gender, time duration between the onset of symptoms and admission to hospital, the history and the symptoms of the patient, signs at presentation, results of the imaging methods, type of surgery, pathology results, length of hospital stay and the outcomes. The present study was approval by Okmeydani Training and Research Hospital Ethics Committee.

28 men and 20 women between ages 16–73 years (mean age 43.1) presented with right iliac fossa pain (Table 1). All patients had localized tenderness leading to a preoperative diagnosis of acute appendicitis. None of the patients applied to the surgery department at the onset of symptoms. They generally preferred self-medication and initial consultation with quacks. Based on our experience in this community, it wasn’t surprising for us to find out at least 4 days between the onset of symptoms and admission to hospital (Table 2).

**Table 1:** Age range of patients (mean 43,1 years)

**Age**	**Number of cases**	**%**
10-20	4	8,3
20-30	8	16,6
30-40	4	8,3
40-50	12	24,9
50-60	12	24,9
>60	8	16,6
Total	48	100

**Table 2:** The time between onset of symptoms and admission to hospital

**Day**	**Number of cases**	**%**
0-1	0	0
1-2	0	0
2-3	0	0
3-4	0	0
4-5	6	12,5
5-6	10	20,8
6-7	18	37,5
>7	14	29,2

The major presenting symptoms were pain in the right iliac fossa in 48 (100%), anorexia in 42 (87,5%), nausea and vomiting in 30 (62,5%), fever in 26 patients (54,2%) (Table 3). When we questioned the patients retrospectively; nausea and vomiting were the major onset symptoms; but the patients could endure this symptoms generally. On physical examination; all patients prefer to lie supine, with the thighs, particularly the right thigh, drawn up; while asked to move, they do so slowly and with caution. Tenderness is at or near the Mc Burney point in 44 (91,6%) patients. Direct rebound tenderness was present at the admission time in 42 patients (87,5%). In addition, referred or indirect rebound tenderness was present in 42 (87,5%) patients. There was a firm, palpable mass in the right iliac fossa in 28 patients (58,3%) (Table 4).

**Table 3:** Major presentation symptoms

**Symptoms**	**Number of cases**	**%**
Pain at the right iliac fossa	48	100
Anorexia	42	87,5
Nausea and vomiting	30	62,5
Fever	26	54,2

**Table 4:** Signs at presentation

**Sign**	**Number of cases**	**%**
Tenderness	44	91,6
Direct rebound	42	87,5
Indirect rebound	42	87,5
Palpable mass	28	58,3

**Figure 1:**
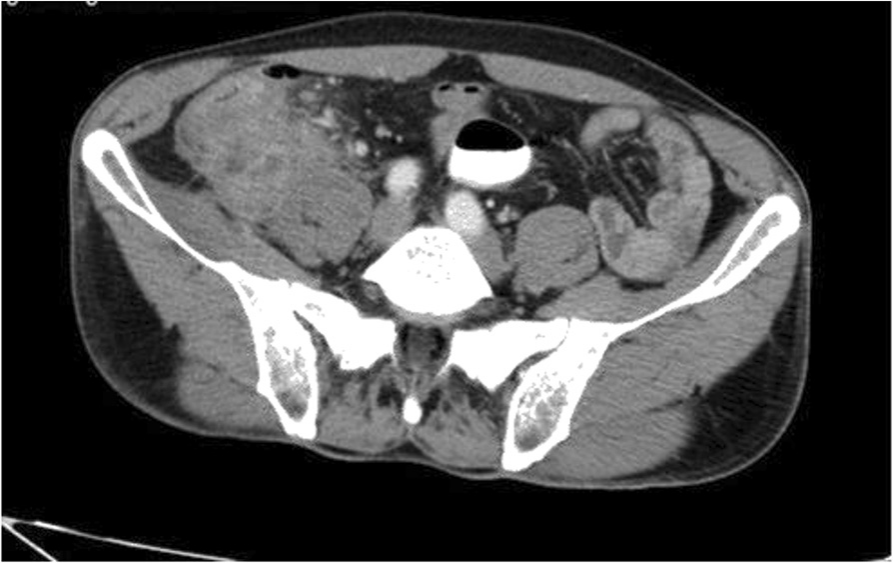
Cecal Diverticulitis: Axial pre-contrast CT image shows mesenteric inflammation adjacent to the distal ileum and cecum, minimal free peritoneal fluid and free air wall thickening and multiple small diverticula in the distal ileum.

**Figure 2:**
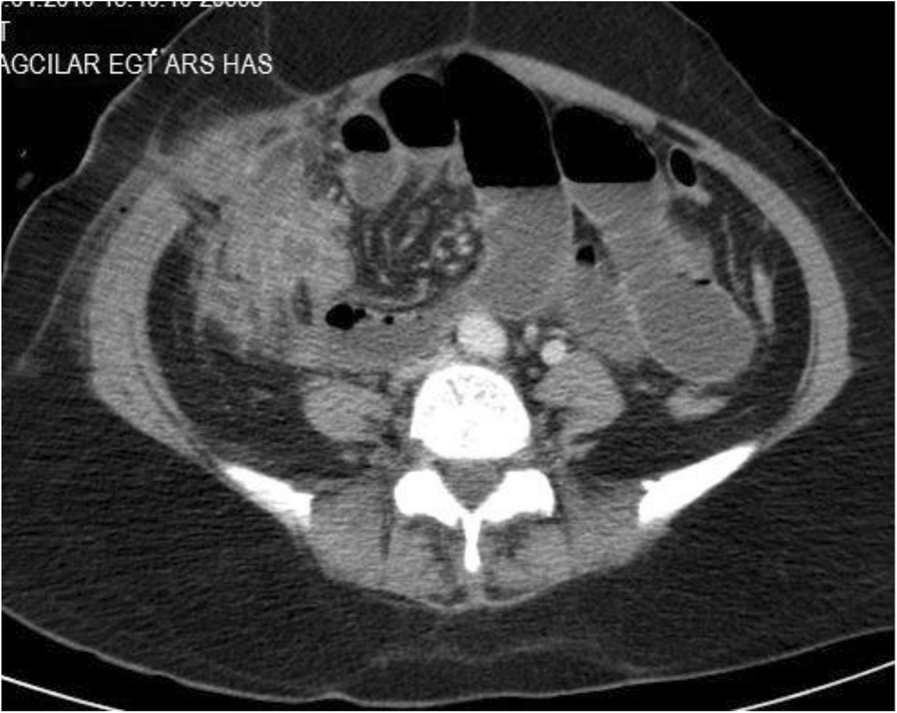
Small bowel and cecal tuberculosis: Contrast-enhanced CT scan shows wall thickening in several distal small bowel loops and cecum.

**Figure 3:**
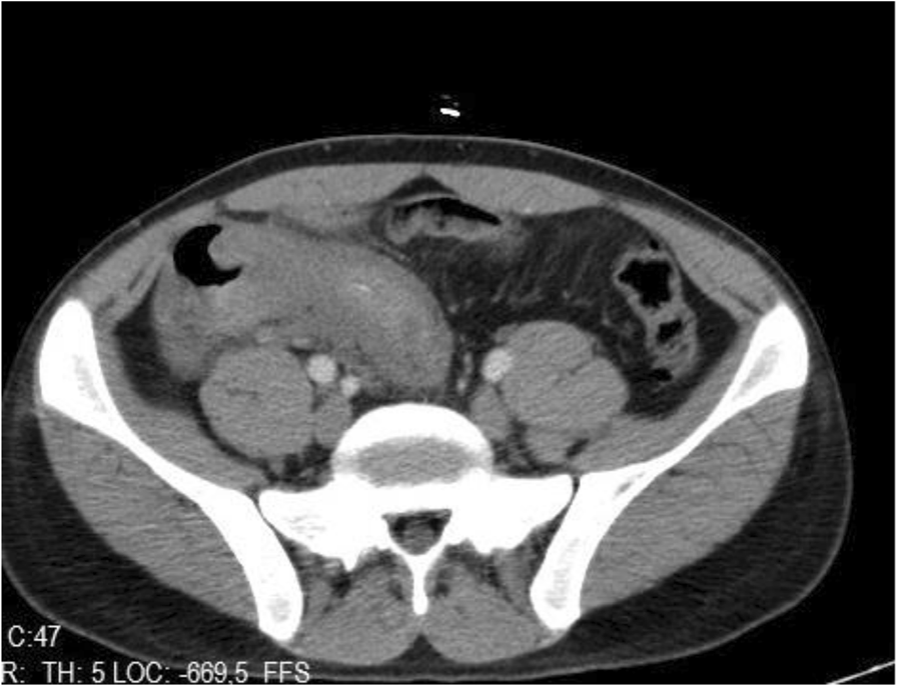
Non-spesific granulomatous: small segment in the terminal ileal wall thickening and inflammation in the adjacent fatty tissue and reactive lymph nodes.

White blood cells were clearly different for each patient. Leucocyte levels ranged between 8.000 to 24.000 and mean level was 16.000 (Table 5). There was no correlation between the onset of symptoms or time of admission to hospital and leucocyte levels. The surgery team preferred abdominal USG and abdominal CT for all patients before the surgery. The scanning methods showed inflammatory cecal masses in all patients, but the radiological team couldn’t decide whether these masses were inflammatory or malignant (Figures 1, 2 and 3). As a result; preoperatively 48 patients (100%) were diagnosed as having appendiceal masses, none of the patients had an appendiceal abscess.

**Table 5:** White blood cell levels

**Leucocyte**	**Number of cases**	**%**
5.000-10.000	4	8,3
10.000-15.000	12	24,9
15.001-20.000	20	41,5
>20.000	12	24,9

**Figure 4:**
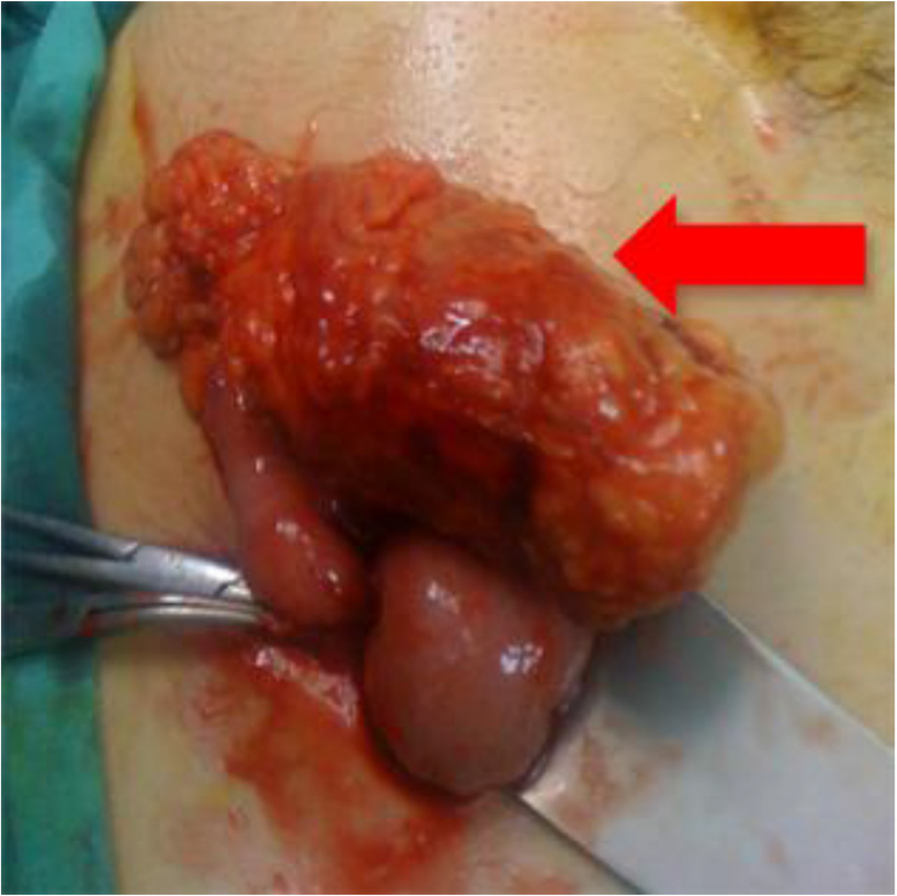
**An unexpected ileocecal mass (red arrow).** Final pathology of the specimen is malign mesenquimal tumor.

After initial laparoscopic exploration ileocecal resection or right hemicolectomy was performed via laparatomy. During the operation, 12 of these patients were suspected to have perforated cecal diverticulitis and underwent ileocecal Resection. 16 patients had an appendicular mass and ileocecal resection was performed because of the uncertainty of the diagnosis and technique difficulties (Figure 4). 4 patients had an appendicular and also cecal rupture in the initial exploration and ileocecal resection performed. In 16 patients malignancy was suspected; in 4 of them right hemicolectomy was performed due to a suspected cecal tumor and in 12 of them the diagnosis remained uncertain, but right hemicolectomy was performed due to the suspicious malignancy. Overall 32 patients underwent ileocecal resection and 16 patients underwent right hemicolectomy. Ileocecal resection was performed through extension of the Mc-Burney incision in 28 patients, but 4 patients had required a separate midline incision because of difficulty of exposure. Right hemicolectomy was performed through conversion to a midline incision in all 16 patients. Primary end-to-side ileocolic anastomosis was performed in all cases.

**Figure 5:**
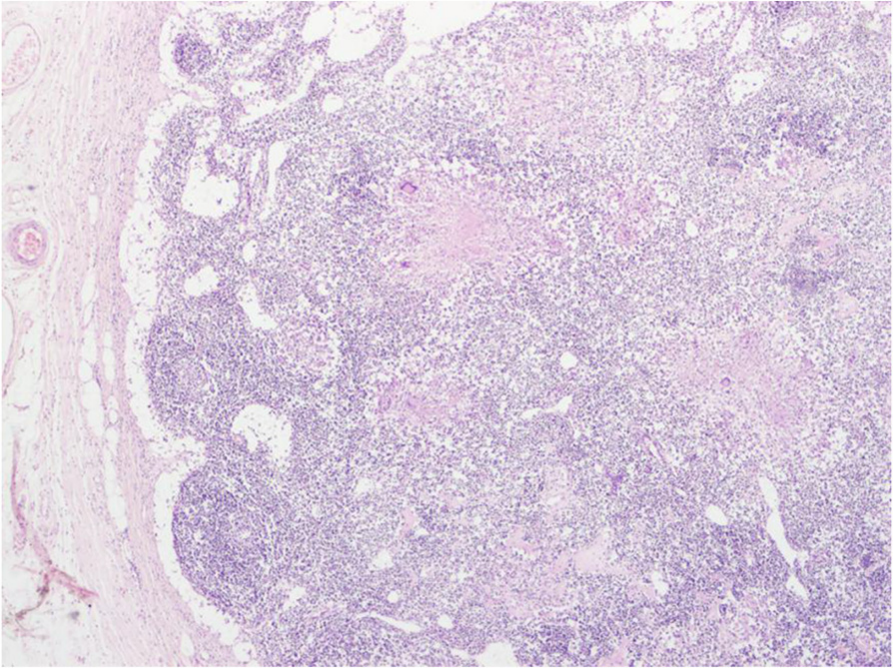
**Ileocecal Tuberculosis.** Tuberculous granulomatous lesions showing caseous necrosis in the centre, and a prominent cuff of lymphocytes and plasma cells at the periphery.

During surgery, the surgeons examined the specimens macroscopically and in 16 patients malignancy was suspected. The histopathologic diagnoses of these patients were tuberculosis in 4, appendiceal phlegmon in 4, non-spesific granulomatous in 2, appendecular endometriosis in 2 and malign mesenquimal neoplasm in 4 patients. Totally the histopathologic diagnosises were as follows, appendiceal phlegmon in 18, perforated cecal diverticulitis in 12, tuberculosis in 6, appendiceal and cecal rupture in 4 patients, malign mesenquimal neoplasm in 4 patients, non-spesific granulomatous in 2 and appendecular endometriosis in 2 patients (Table 6) (Figure 5).

**Table 6:** The final pathology

**Findings**	**Number of cases**	**%**
Appendiceal phlegmon	18	37,5
Perforated cecal diverticulitis	12	25,0
Tuberculosis	6	12,5
Appendiceal-cecal rupture	4	8,3
Malign mesenquimal neoplasm	4	8,3
Non-spesific granulomatous	2	4,2
Appendecular endometriosis	2	4,2

There was no mortality and all of the patients were discharged in good health. There was only one complication of wound infection. The postoperative hospital stay duration was between 1 to 7 days, especially depending on the co-morbidity of the patients.

## Discussion

Appendicitis is the most common cause of acute abdomen requiring emergency surgery. Only half of the patients present classical clinical diagnosis of appendix infection [1]. Sometimes inflammatory cecal masses or cancers mimick acute appendicitis and during the operation the surgeons can not distinguish the pathology. Inflammation and cancer frequently form masses which are hardly distinguishable, and surgeons are often challenged to determine the pathologic origin of an inflammatory mass. Such masses involving the cecum are relatively uncommon when one excludes those resulting from appendicitis. Because such lesions are rare they are often reported, many are found unexpectedly at emergency operations as lesions simulating appendicitis [9].

Although most of the appendicular masses are benign and can be solved simplistically, a number of other conditions, some of them sinister, can be a dilemma for the surgeons. Such conditions including cecal diverticulitis, cecal carcinoma, ileocecal tuberculosis, non-specific granulomatous, appendicular endometriosis are more complex and should be managed and treated carefully. Sometimes in the emergency conditions the surgeon could not decide the exact diagnose and exclude malignancy. In our study, we could not exclude malignancy in 16 patients during the operative period.

Ultrasonography has been advocated as the diagnostic modality of choice, revealing the diagnosis in%72 of cases, but computerized tomography (CT) scan is superior [10]. In our experience we saw that ultrasonography could not guide us for the diagnosis in majority of the patients. We suggest that in overdue and suspicious cases CT should be the first choice for the diagnosis. Most of the authors described the relation between the leukogram and acute abdomen. We could not observe any correlation between onset of symptoms or the time of admission to hospital and laboratory tests especially leucocyte levels.

Some management issues has been surrounded with controversy with no general agreement among surgeons; a recent questionnaire study of 67 consultant and specialist register surgeons in the Mid-Trent region of England showed no agreed consensus on the management of appendiceal mass [11]. Most inflammatory cecal masses are due to benign pathologies and could be managed safely and sufficiently with ileocecal resection. Careful intraoperative assessment including examination of the resected specimen is essential to exclude malignancy, which would require right hemicolectomy [8-11]. In the present study, overall 32 patients underwent ileocecal resection and 16 patients underwent right hemicolectomy. 4 of the right hemicolectomies were performed for cecal tumor while 12 of them were performed for the suspicious malignancy. No malignancy was determined in these 12 patients.

Based on our experience in this community, it wasn’t surprising that none of the patients admitted to hospital before 4 days after the onset of symptoms. Delayed admission to the hospital is common in our rural hospitals. It depends on numerous factors. Self-medication, especially anti-pyretics and analgesics is the most common one. Poverty, illiteracy, absence of health insurance and phobias are mainly responsible for the community indulging in self-medication. This postponement in admission to hospital by rural dwellers appears to be a common problem in most rural communities in the world. Harouna et al. [12] in a study of the current prognosis of appendicitis in the Niger Republic in 2000 discussed this point and emphasized the deterioration of services offered by state health structures as one of the banes of health care services in Africa. The surgeons that work in rural hospitals should be aware of these delayed presentations. If a surgeon evaluates the case in emergency conditions as acute abdomen and cannot diagnosis the condition definitely, ileocecal and right hemicolectomy can be performed as a first choice for the suspicious malignancy.

## Conclusions

Most inflammatory masses are caused by benign pathologies, and usually ileocecal resection is the procedure of choice. Rarely, when surgeons can not determine the pathology clearly and suspect malignancy they can prefer to perform right hemicolectomy or ileocecal resection. Because of the high incidence of appendiceal mass in our rural community, there is a need for all concerned to make sincere efforts to lower these figures.

### Consent

Written informed consent was obtained from the patient for publication of this care report and any accompanying images. A copy of the written consent is available for review by the Editor-in-Chief of this journal.

## Competing interests

The authors declare that they have no competing interests.

## Authors’ contributions

HG and BK took care of patient and wrote the initial draft. HG, BK, FS and GA operated the patent. BK, GA and IAB edited manuscript with literature review. All authors read and approved the final manuscript.
